# Weight regain following discontinuation of glucagon-like peptide-1 receptor agonists in adults who are overweight or obese: a systematic review and meta-analysis

**DOI:** 10.7717/peerj.21713

**Published:** 2026-09-08

**Authors:** Huaji Qi, Qiang Gao, Haiyan Chen, Xiaodan Du, Bobo Pan

**Affiliations:** 1 Hangzhou Linping District Integrated Traditional Chinese and Western Medicine Hospital, Hangzhou, Zhejiang, China; 2 Pharmacy Department, Ningbo No.2 Hospital, Wenzhou Medical University, Ningbo, Zhejiang, China

**Keywords:** GLP-1RA, Tirzepatide, Semaglutide, Weight regain

## Abstract

**Objective:**

This study aims to explore the effects of glucagon-like peptide-1 receptor agonists (GLP-1RAs) on weight changes and the occurrence of adverse reactions in overweight or obese adults after drug withdrawal.

**Methods:**

Computerized searches were conducted in evidence-based databases such as PubMed, Embase, Cochrane Library and Scopus. The search period was from the establishment of the database to December 2025. Collect randomised controlled trials (RCTs) and controlled trials on GLP-1RAs, including tirzepatide, semaglutide, liraglutide, and dulaglutide, for the treatment of overweight or obese adult patients. The risk of bias in the included studies was assessed using the Cochrane Risk of Bias V2.0 tool provided by the Cochrane Collaboration, and meta-analysis was performed using the R programming language.

**Results:**

A total of 699 studies were initially retrieved. Eventually, six studies involving 8,993 patients were included in the quantitative analysis, comprising 5,553 patients in the discontinuation group and 3,440 in the continued treatment group. The results of the meta-analysis showed that, compared with the continued treatment group, the weight difference in the discontinuation group was mean difference (MD) = 17.90%, 95% confidence interval (CI) [14.11–21.69], *P* < 0.0001. It can be seen that there was a significant rebound in weight after drug withdrawal, and there was statistical heterogeneity among the studies (*P* = 0.0082). Subgroup analysis further revealed that the weight rebound amplitude after discontinuation of tirzepatide was significantly higher than that of semaglutide. This result suggests that the differences in the mechanism of action of different GLP-1RAs may be the reason for the differences in weight changes after discontinuation. In addition, the percentage difference in body weight between after and before drug withdrawal was MD = 9.11%, 95% CI [7.91–10.30], *P* < 0.0001, further verifying the trend of weight rebound after drug withdrawal. The summary of adverse reaction reports analyzed and studied indicates that after drug withdrawal, the overall adverse reactions of patients decreased, gastrointestinal adverse reactions decreased, and the incidence of cardiovascular events was not affected by drug withdrawal.

**Conclusion:**

There is a significant weight rebound phenomenon after discontinuation of GLP-1RAs, and the rebound magnitudes vary among different types of drugs. At the same time, there is a risk of adverse reactions during the use of such drugs.

## Introduction

Obesity is a chronic metabolic disorder involving genetic predisposition, metabolic dysfunction, environmental factors, and unhealthy dietary behaviours. It constitutes an independent risk factor for non-communicable diseases such as cardiovascular disease, type 2 diabetes, and certain malignancies (Powell-Wiley et al., 2021; Wharton et al., 2020). The 2025 World Obesity Atlas projects that the global adult obesity population will rise to 1.13 billion by 2030 (World Obesity Federation, 2025). The impact of weight cycling on health has long been controversial. Traditional views suggest that repeated weight loss and regain may increase the risk of adverse health outcomes (Gaesser et al., 2025); however, recent evidence indicates that the benefits of weight loss, such as improvements in metabolic parameters, cardiovascular health, and quality of life, generally outweigh the potential risks associated with weight fluctuations, and the harms of sustained obesity far exceed those of weight cycling itself (Magkos & Stefan, 2026).

Glucagon-like peptide-1 receptor agonists (GLP-1RAs) promote weight loss through multiple mechanisms, including appetite suppression, delayed gastric emptying, and modulation of hypothalamic satiety signalling pathways (Kim et al., 2024; Li et al., 2024). Clinical studies confirm that overweight or obese patients treated with semaglutide 2.4 mg weekly achieve significant weight reduction of 14.9% (Wilding et al., 2021). Tirzepatide is a dual agonist of both GLP-1 and glucose-dependent insulinotropic polypeptide (GIP) receptors, also termed a GIP/GLP-1 receptor agonist. The mechanism of action of GIP resembles that of GLP-1RAs. Studies indicate that obese patients treated with the maximum dose of tirzepatide (15 mg weekly) achieved an average weight reduction of 20.9% (Jastreboff et al., 2022). Furthermore, GLP-1RAs demonstrate significant clinical benefits in improving glycemic control, sustaining weight loss, and reducing cardiovascular event risk (Lincoff et al., 2023; Garvey et al., 2023). However, rapid weight regain occurs after discontinuation of modern incretin-based medications, with studies showing that approximately 55%–65% of the weight lost is regained within one year of discontinuation (Gaesser et al., 2025). This phenomenon has sparked discussions regarding intermittent use of these agents. Meanwhile, factors such as high treatment costs and gastrointestinal adverse effects necessitate premature treatment discontinuation in some patients (Wilding et al., 2021; Moiz et al., 2024; le Roux et al., 2023).

Current follow-up data regarding post-treatment weight changes in patients discontinuing GLP-1RAs remain limited, with considerable heterogeneity observed across studies (Jensen et al., 2024). Existing systematic reviews assessing weight regain after discontinuing GLP-1RAs indicate considerable variation in follow-up durations across included studies, and the study populations were not strictly confined to discontinuation cohorts following weight loss induced by GLP-1RAs or GIP/GLP-1RAs (Berg et al., 2025; Tzang et al., 2025). Furthermore, the number of randomised controlled trials (RCTs) evaluating post-discontinuation weight rebound remains insufficient, and current clinical guidelines offer no specific recommendations regarding dose-tapering regimens prior to discontinuation or structured weight maintenance strategies.

Therefore, this systematic review and meta-analysis aims to: (1) Quantitatively assess the magnitude of weight change in patients 48 weeks after discontinuing GLP-1RAs or GIP/GLP-1RAs; (2) Analyse differences in weight rebound patterns between different GLP-1RAs; (3) Directly compare the difference between post-discontinuation weight and pre-discontinuation baseline weight, quantifying the absolute rebound effect. This study strictly restricted inclusion to individuals who achieved weight loss through GLP-1RAs or GIP/GLP-1RAs treatment before discontinuation. The findings will provide evidence-based guidance for optimising weight management strategies in overweight or obese patients and offer scientific direction for developing sustainable long-term weight management programmes for this population.

## Data and Methods

### Inclusion and exclusion criteria

Study population: All included studies involved adult overweight or obese patients, defined as follows: aged ≥ 18 years, with a body mass index (BMI) >30 kg/m^2^ or a BMI ≥ 27 kg/m^2^ accompanied by at least one weight-related comorbidity, with or without diabetes.

Study types: Only Randomized Controlled Trials (RCTs) and controlled trials were included.

Interventions: All studies were required to include at least one intervention group: discontinuation of GLP-1RAs or GIP/GLP-1RAs following weight loss induction, with a follow-up period ≥ 48 weeks (calculated from discontinuation). RCTs require a control group, whereas single-arm studies generally do not include a control group. Studies must report routine post-intervention metrics such as weight loss relative to baseline and gastrointestinal adverse reactions.

Outcome measures: Primary outcome—Weight gain after discontinuation of GLP-1RA (percentage increase or decrease relative to baseline). Secondary outcome—adverse reactions following discontinuation.

Exclusion criteria: ① Subjects who are not overweight; or subjects being animals; ② Intervention methods other than GLP-1RAs; ③ Investigations, case analyses, reviews, protocols, pilot studies, *etc.*; observational cohort studies, case-control studies; ④ Studies lacking outcome measures, where data are unavailable or cannot be converted for use.

### Databases and search strategy

Databases searched: PubMed, Embase, Cochrane Library and Scopus. Studies were eligible for inclusion if published from the inception of the database up to 31 December 2025. Search terms: “GLP-1 receptor agonist”, “GLP-1RA”, “tirzepatide”, “semaglutide”, “liraglutide”, “dulaglutide”, “withdrawal”, “discontinuation”, “weight regain”. Searches were conducted using free-text queries.

### Studies screening and quality assessment

Following study retrieval, duplicate records were excluded using software. Two researchers screened titles and abstracts against inclusion and exclusion criteria. Full texts of remaining studies were obtained; where online access was unavailable, authors were contacted. Full-text articles were then reviewed for further selection. The Cochrane Risk of Bias Tool V2.0 (Cochrane Collaboration) was employed to assess risk of bias across five domains for included studies, with each domain rated as “low”, “some concern of risk” or “high”.

### Data extraction

Studies data were independently extracted by two researchers, Huaji Qi and Qiang Gao, including the authors of the studies, publication date, grouping method, number of cases in each group, patient age, gender ratio, intervention measures, follow-up time, outcome indicators, and corresponding data. After completing the data extraction, the two researchers cross-checked each other’s results and discussed the discrepancies. In case of inconsistency, they conducted joint discussions and negotiations. If a consensus still could not be reached, the corresponding author, Bobo Pan, made the final decision through arbitration.

### Statistical analysis

Analysis was conducted using the “meta” package in R (version 4.4.1). Comparisons of pre- and post-intervention body weight, and between the discontinuation and continuation groups, were assessed using the mean difference (MD). Comparisons of efficacy and adverse event incidence between groups post-intervention were assessed using the risk ratio (RR). Heterogeneity among studies was assessed using I^2^ analysis and Q statistics, with I^2^ > 50% or *P* < 0.1 indicating significant heterogeneity. Subgroup analyses were conducted to explore sources of heterogeneity. Fixed-effects modelling (Mantel-Haenszel) was applied when no heterogeneity was present, while the Der Simonian-Laird method was used when heterogeneity existed. A *P*-value < 0.05 indicated a statistically significant combined effect size. Evidence quality was assessed using GRADE pro software and graded as high, moderate, low, or very low. This systematic review and meta-analysis were prospectively registered on PROSPERO (CRD 420261320886) and conducted in accordance with the PRISMA guidelines.

## Results

### Studies screening results

**Figure 1:**
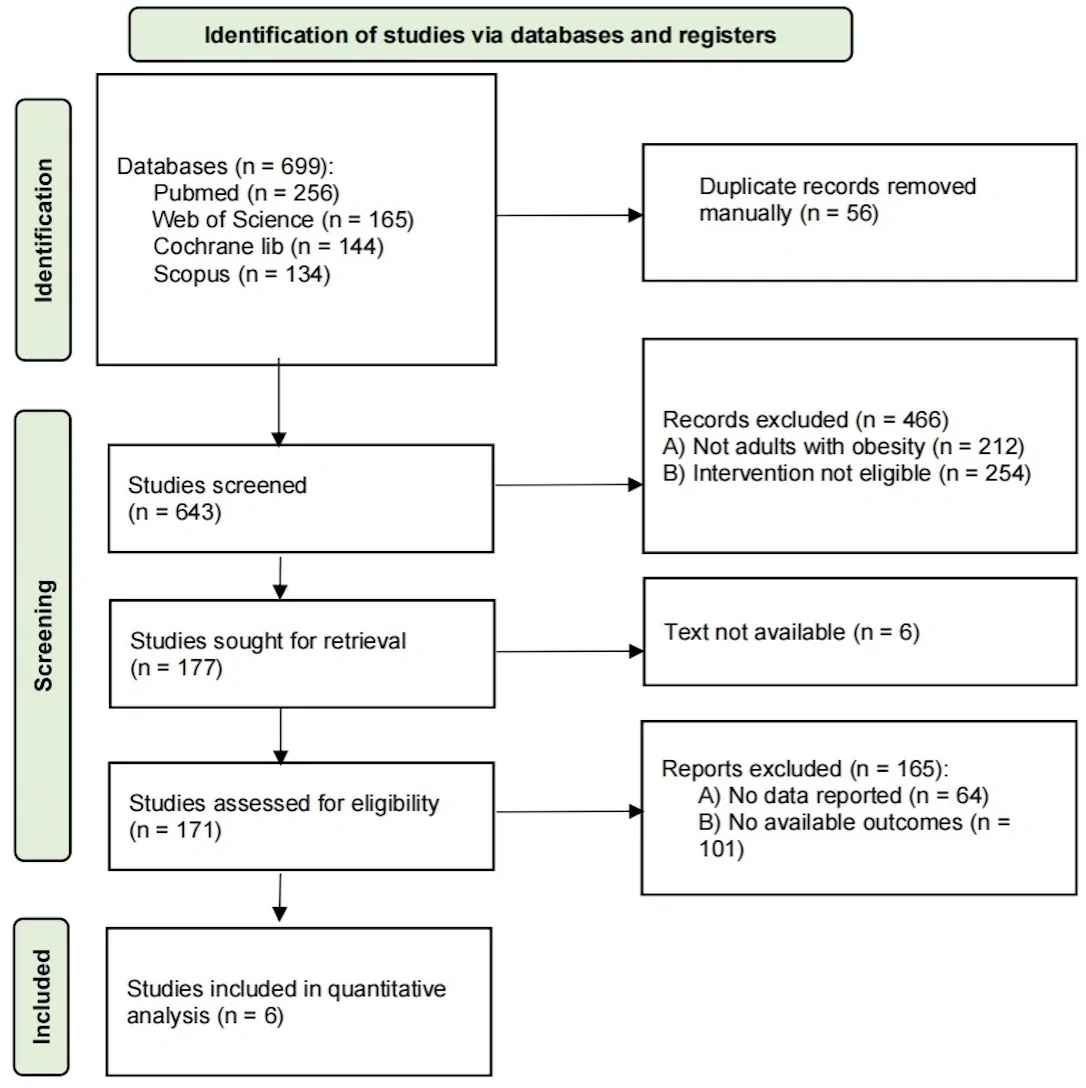
Study selection flowchart.

Figure 1 depicts the studies selection flowchart. An initial search yielded 699 publications. Following deduplication and screening, six studies were ultimately included in the quantitative analysis (Rubino et al., 2021; Wilding et al., 2022; Wilkinson et al., 2023; Gasoyan et al., 2025b; Aronne et al., 2024; Jensterle, Ferjan & Janez, 2024). Kubota, Yamamoto & Yoshiyama (2023) included a case series, but the study design was not a controlled study; Gibble et al. (2025) was excluded as it was one of multiple analyses from the same trial. Maya et al. (2025) observed effects of discontinuing GLP-1RAs on pregnancy outcomes but it was excluded as an observational study. In addition, although liraglutide and dulaglutide were searched, no studies meeting the inclusion criteria (follow-up ≥ 48 weeks after discontinuation and study design as RCT or controlled trial) were identified.

### Study characteristics

The fundamental characteristics, interventions, and outcome measures of all included studies are summarised in Table 1. A total of 8,993 patients were enrolled, comprising 5,553 who discontinued GLP-1RAs therapy and 3,440 who received continuous maintenance treatment. The six included studies were published between 2021 and 2025. Five RCTs and one single-arm controlled study were included. The GLP-1RAs covered in the included studies comprised semaglutide and tirzepatide.

**Table 1:** Basic characteristics, interventions, and outcome measures of included studies.

Author	Publi cation year	Study design	Country	Population	Age (years)	BMI, mean (SD), kg/m^2^	Sex	Sample size (Discontinued/ Maintained)	Intervention (glp-1 receptor agonist)	Behavioral intervention	Control	Follow-up
Rubino et al. (2021)	2021	RCT	10 countries	Adults with overweight/obesity (BMI ≥30, or ≥27 with ≥1 weight-related comorbidity) without diabetes (HbA1c < 6.5%)	46 (12)	38.4 (6.9)	79% Female	268/535	Semaglutide 2.4 mg	Reduced-calorie diet and physical activity	Placebo	48 weeks
Wilding et al. (2022)	2022	RCT Extension	Multinational	Adults with overweight/obesity (BMI ≥30, or ≥27 with ≥1 weight-related comorbidity) without diabetes (HbA1c < 6.5%)	49 (12)	37.6 (7.5)	67% Female	327/0	Semaglutide 2.4 mg	Lifestyle intervention was withdrawn	Placebo	52 weeks
Wilkinson et al. (2023)	2023	*Post hoc* analysis	Multinational	White or Black adults with overweight/obesity without diabetes (from STEP 4 )	47 (12)	38.5 (7.0)	79% Female	224/467	Semaglutide 2.4 mg	Reduced-calorie diet and physical activity	Placebo	68 weeks/104 weeks
Gasoyan et al. (2025b)	2025	Quasi-RCT	USA	Adults with overweight/obesity without type 2 diabetes	51.3 (13.4)	39.7 (7.8)	75.2% Female	3,401/1,629 (Sema) 973/474 (Tirz)	High: Semaglutide 1.7/2.0/2.4 mg; Tirzepatide 10/12.5/15 mg; Low: All other doses	Unknown	Placebo	52 weeks
Aronne et al. (2024)	2024	RCT	4 countries	Adults with overweight/obesity (BMI ≥30, or ≥27 with ≥1 weight-related complication) without diabetes	48 (13)	38.4 (6.6)	70.6% Female	335/335	Tirzepatide 10/15 mg	Diet and physical activity	Placebo	52 weeks
Jensterle, Ferjan & Janez (2024)	2024	Single-Arm controlled study	Slovenia	Obese women with PCOS without diabetes	33.7 (5.3)	36.4 (33.2–38.6)	100% Female	25/0	Semaglutide 1.0 mg	Promotion of healthy lifestyle was continued	N/A	2 years

### Assessment of study quality and bias

**Figure 2:**
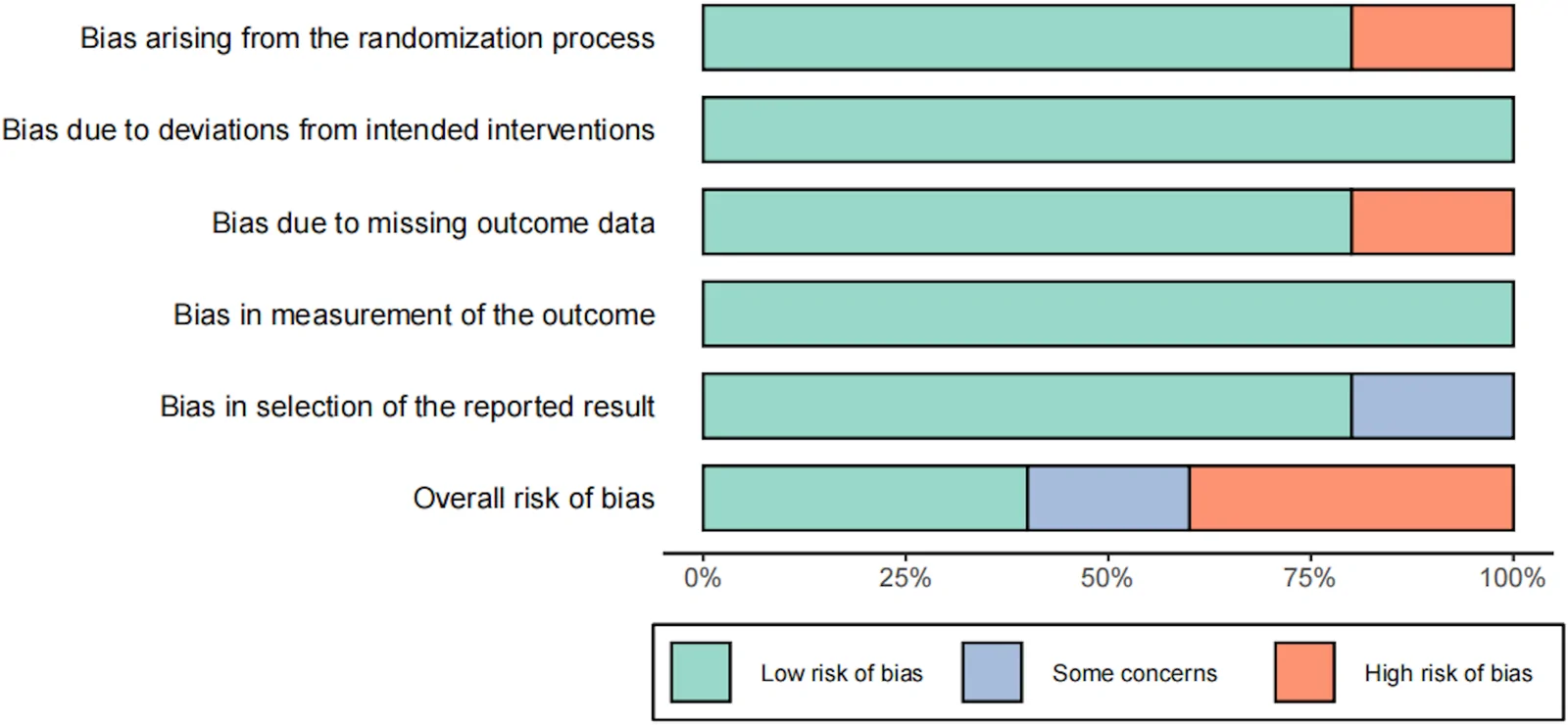
Overall studies risk map.

For the five included studies (Rubino et al., 2021; Wilding et al., 2022; Wilkinson et al., 2023; Gasoyan et al., 2025b; Aronne et al., 2024), a risk of bias assessment based on the Cochrane ROB 2.0 tool was conducted, with results presented in Table 2. Among these, the study by Wilding et al. (2022) included only approximately 17% of the original participants in the extension phase, leading to substantial missing data and selection bias. The study by Gasoyan et al. (2025b) exhibited a high risk of bias with respect to the randomization process. The overall risk of bias is illustrated in Fig. 2.

### Pooled effect size results

#### Weight rebound following discontinuation *versus* continued medication

**Figure 3:**
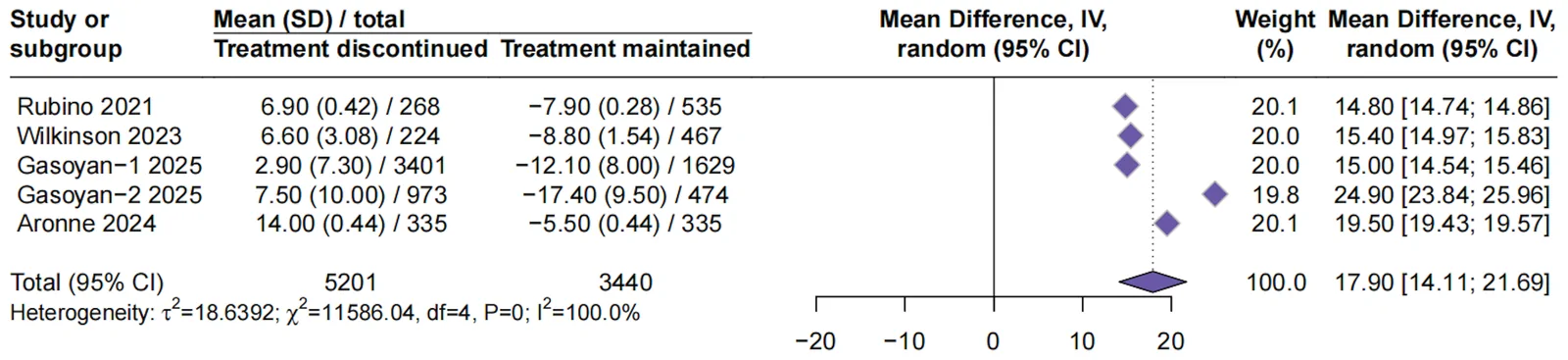
Weight gain between discontinuation and continuation of medication.

All four studies reported weight comparisons between discontinuation and continuation interventions. The intervention group comprised 5,201 patients, while the control group included 3,440 patients. Significant statistical heterogeneity was observed between studies (*I* ^2^= 100%, Q-test *P* < 0.01). Using a random-effects model, the weight difference between discontinuation and continuation interventions was calculated as (MD = 17.90%, 95% CI [14.11–21.69], *P* < 0.0001), as detailed in Fig. 3.

**Table 2:** Risk of bias assessment based on ROB 2.0.

Study	Randomisation process	Deviations from intended interventions	Missing outcome data	Measurement of the outcome	Selection of the reported result	Overall bias	Weight
Rubino et al. (2021)	Low	Low	Low	Low	Low	Low	20
Wilding et al. (2022)	Low	Low	High	Low	Low	High	20
Wilkinson et al. (2023)	Low	Low	Low	Low	Some concerns	Some concerns	20
Gasoyan et al. (2025b)	High	Low	Low	Low	Low	High	20
Aronne et al. (2024)	Low	Low	Low	Low	Low	Low	20

**Notes.**

Potential risks correspond to “Some concerns of risk” in English, “Low” risk corresponds to “Low risk”, and “High” risk corresponds to “High risk”.

#### Subgroup analysis by drug type

**Figure 4:**
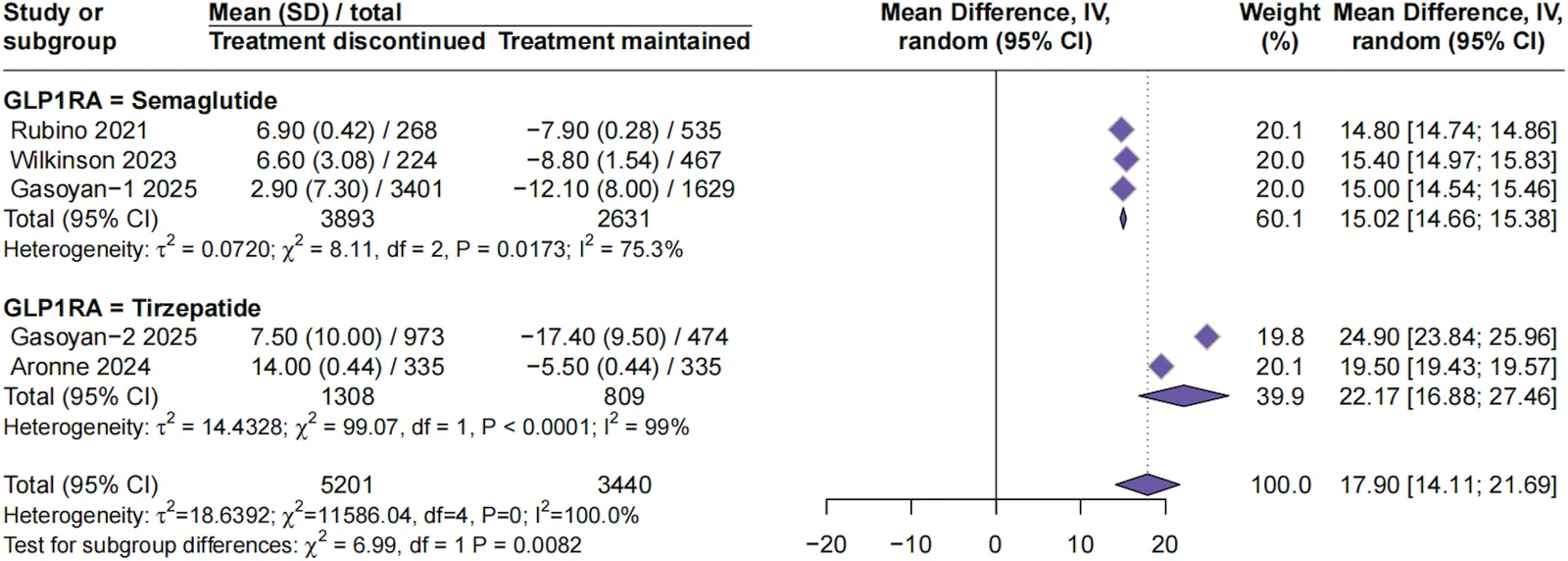
Subgroup analysis by drug class.

Subgroup analysis based on drug type was conducted across the five studies (Fig. 4), revealing significant differences between subgroups (*P* = 0.0082); weight rebound following discontinuation of tirzepatide was markedly higher than that observed with semaglutide.

#### Summary of adverse reaction reports

All five studies reported the incidence of adverse reactions in patients following intervention. The meta-analysis results are presented in Table 3.

### GRADE evidence quality assessment

Evidence quality was assessed using the GRADE framework (Table 4). The primary outcome showed a 17.90% weight regain in the discontinuation group compared to the continuous treatment group (95% CI [14.11–21.69]), with very low certainty of evidence. Subgroup analysis indicated low certainty of evidence for the Semaglutide group, while the tirzepatide group exhibited greater rebound after discontinuation (MD = 22.17% *vs* 15.02%, *P* = 0.0082), though evidence quality was very low. Furthermore, comparison before and after discontinuation showed a 9.11% weight gain (95% CI [7.91–10.30]), with very low evidence quality. Secondary outcomes included reduced overall adverse events (RR = 0.77) and gastrointestinal events (RR = 0.39) upon discontinuation, with high and moderate quality evidence, respectively; no difference in cardiovascular events (RR = 0.87) was observed, with very low quality evidence.

**Table 3:** Meta-analysis results for adverse reaction outcomes.

Outcomes	Number of studies	Analysis model	*I*^2^ and Cochran’s Q *P*-value	Effect size type	Pooled effect size	*P*-value
Any events	2	Common effect model	0% with *P* = 0.431	OR	0.7681 [0.6092–0.9684]	0.0256
Gastrointestinal disorders	2	Random effects model	69.8% with *P* = 0.068	OR	0.3888 [0.2346–0.6443]	0.0002
Cardiovascular disorders	2	Random effects model	71% with *P* = 0.063	OR	0.8706 [0.0587–12.9049]	0.9198

**Table 4:** GRADE assessment.

Outcome	Effect size (95% CI)	Number of studies	Sample size	Risk of bias	Heterogeneity	Precision	Indirectness	Publication bias	Final level of evidence
Post-treatment weight regain	MD 17.90% (14.11–21.69)	4	8,641	Decreased by 1 grade	Decreased by 2 levels	No downgrade	No downgrade	No downgrade	Very low
Semaglutide subgroup	MD 15.02% (14.66–15.38)	3	6,524	Decreased by 1 level	Decreased by 1 grade	No downgrade	No downgrade	No downgrade	Low
Tirzepatide subgroup	MD 22.17% (16.88–27.46)	2	2,117	Decreased by 1 grade	Decrease by 2 grades	No downgrade	No downgrade	No downgrade	Very low
Weight rebound after discontinuation compared to before discontinuation	MD 9.11% (7.91–10.30)	2	704	Decreased by 1 grade	Decreased by 2 grades	No downgrade	No downgrade	No downgrade	Very low
Overall adverse events	OR 0.77 (0.61–0.97)	2	1,473	No downgrading	No downgrading	No downgrade	No downgrade	No downgrade	High
Gastrointestinal adverse events	OR 0.39 (0.23–0.64)	2	1,473	No downgrade	Decrease by 1 level	No downgrade	No downgrade	No downgrade	Moderate
Cardiovascular adverse events	OR 0.87 (0.06–12.90)	2	1,473	No downgrade	Decreased by 1 grade	Downgrade by 2 grades	No downgrade	No downgrade	Very low

## Discussion

This study synthesised data from six trials involving 8,993 patients. Meta-analysis confirmed key clinical outcomes following discontinuation of GLP-1RAs and GIP/GLP-1 dual receptor agonists: compared with continued treatment, discontinuation groups exhibited significant weight regain (MD = 17.90%, 95% CI [14.11–21.69], *P* < 0.0001), with a 9.11% increase in weight post-discontinuation relative to pre-discontinuation levels (MD = 9.11%, 95% CI [7.91–10.30], *P* < 0.0001) compared to the continued treatment group. Subgroup analysis further revealed that the magnitude of weight regain following tirzepatide discontinuation was significantly greater than that following semaglutide discontinuation (*P* = 0.0082). With regard to safety, overall and gastrointestinal adverse events decreased after treatment cessation, while the incidence of cardiovascular events showed no significant change. However, due to the risk of bias and heterogeneity among the included studies, the GRADE assessment indicated that the quality of evidence ranged from low to very low.

Although GLP-1 weight loss drugs have significant weight loss effects, statistics show that a weight loss of 5% to 10% can improve type 2 diabetes and related complications (Gasoyan et al., 2025a). However, continued use of GLP-1RAs may be constrained by tolerability, efficacy, accessibility, and healthcare reimbursement limitations. Patients frequently discontinue treatment within one year, facing weight rebound after cessation of GLP-1RAs (Rodriguez et al., 2025; Prillaman, 2024).

During GLP-1 receptor agonist therapy, appetite is suppressed, and dietary behaviour is improved *via* central and peripheral pathways, helping to sustain reduced body weight (Friedrichsen et al., 2021; Jensen et al., 2022; Maselli et al., 2022). Following discontinuation, the drug’s core effects cease, releasing the inhibitory influence on the hypothalamic appetite centre. Levels of appetite-regulating hormones such as leptin and ghrelin gradually revert to pre-treatment states. The body is unable to autonomously sustain the metabolic equilibrium achieved during treatment, consequently attempting to restore the original weight set point through increased food intake and reduced energy expenditure. This represents the primary mechanism driving rapid weight rebound (Iepsen et al., 2015; Gilden, Catenacci & Taormina, 2024; Sumithran et al., 2011). The subgroup analysis demonstrated that the magnitude of weight regain following tirzepatide discontinuation was significantly greater than that observed with semaglutide (*P* = 0.0082). In the STEP 1 extension study, semaglutide treatment over 68 weeks resulted in a 17.3% weight reduction, which rebounded by 11.6 percentage points after withdrawal, yielding a long-term net weight loss of only 5.6% (Wilding et al., 2022). In the SURMOUNT-4 study, tirzepatide treatment over 36 weeks produced a 20.9% weight reduction, followed by a 14.0% rebound upon discontinuation, with the net weight loss at week 88 declining to approximately 9.9% (Aronne et al., 2024). West et al. (2026) suggested that, on the one hand, greater initial weight loss is generally associated with more rapid weight regain after discontinuation, so the more pronounced rebound in the tirzepatide group may be related to its greater initial weight reduction; on the other hand, pharmacotherapy reduces the difficulty of behavioral modification by suppressing appetite and enhancing satiety, which may undermine patients’ internalization of conscious dietary control and physical activity efforts, and the lack of autonomous behavioral capacity for weight maintenance after drug cessation may further exacerbate weight regain. Furthermore, from the perspective of pharmacological mechanisms, the difference in weight regain between tirzepatide and semaglutide may also be related to their distinct receptor profiles. Tirzepatide is a dual GIP/GLP-1 receptor agonist that not only suppresses appetite and delays gastric emptying through the GLP-1 pathway, but also activates the GIP receptor to enhance lipolysis and improve insulin sensitivity (Cho, Lee & Jung, 2023). It is therefore hypothesized that, upon drug discontinuation, the disruption of the dual metabolic adaptive state and the simultaneous withdrawal of both pathways may trigger a more pronounced compensatory response, leading to greater weight regain. In contrast, semaglutide acts solely on the GLP-1 receptor, with a relatively limited scope of metabolic intervention; thus, the metabolic perturbation following discontinuation is milder, and the compensatory response is correspondingly less intense. These mechanistic hypotheses warrant further experimental investigation to be validated.

The adverse reaction reports summarised in this analysis indicate a general reduction in adverse events following discontinuation, particularly gastrointestinal complaints, while cardiovascular event rates remain unaffected by cessation. Common adverse reactions to GLP-1RAs include gastrointestinal disturbances such as nausea, diarrhoea, or constipation; these naturally resolve upon discontinuation as the drug’s gastrointestinal irritation ceases (Aronne et al., 2021; Chen et al., 2024). Research indicates that cardiovascular benefits may persist for some time after discontinuing liraglutide, suggesting short-term cessation does not immediately increase cardiovascular risk (le Roux et al., 2017). However, the metabolic benefits derived from weight loss also diminished after drug discontinuation, with cardiometabolic parameters that had improved during treatment largely returning to baseline levels, suggesting that both weight loss and metabolic benefits are highly dependent on continued pharmacotherapy (Quarenghi et al., 2025). The 17.9% weight regain observed in the present study further corroborates this pattern, indicating that the weight loss benefits achieved during treatment may be attenuated or even completely offset, along with the risk of reversing the improvements in cardiometabolic outcomes. However, it should be noted that this study did not include cardiovascular outcome data from long-term discontinuation (>1 year), and long-term risks require further validation.

The 17.90% weight regain observed in the present study is consistent with the findings of other independent studies and reviews. West et al. (2026) further quantified this trend, reporting that the monthly rate of weight regain for novel incretin mimetics such as semaglutide and tirzepatide can reach 0.8 kg, with body weight returning to baseline approximately 1.5 years after drug discontinuation, and that the rate of regain is faster than that following behavioral intervention alone. Real-world studies likewise support this pattern; a cohort study of 125,474 patients in the United States demonstrated that for every 1% weight regain following discontinuation of GLP-1RAs, the risk of restarting treatment increased by 2.3% to 2.8%, indirectly reflecting the prevalence of rebound weight gain (Rodriguez et al., 2025). A post-hoc analysis of the SURMOUNT-4 trial (Horn et al., 2026) demonstrated that among obese patients achieving ≥10% weight loss after 36 weeks of tirzepatide treatment, the majority experienced ≥25% weight regain within 52 weeks of discontinuation. Waist circumference increase correlated with the extent of weight regain; greater regain was associated with more pronounced reversal of cardiovascular metabolic improvements, such as waist circumference, blood pressure, lipid profile, and glycated haemoglobin. Only patients with <25% regain maintained benefits in central adiposity. Cardiovascular and metabolic benefits are lost when weight regain exceeds 25% of initial weight loss, consistent with findings from studies including the Aronne and Look Ahead trials (Aronne et al., 2021; Berger et al., 2019). Concurrently, studies in patients with non-alcoholic fatty liver disease (NAFLD) indicate that discontinuation of liraglutide resulted in 1.8 kg weight regain, alongside increases in liver fat fraction (LFF) and the hepatocyte apoptosis marker c-c*κ*B-18, suggesting that metabolic benefits are highly dependent on sustained medication (Khoo et al., 2019).

This study included 6 studies, comprising one single-arm study, with a total sample size of 8,993 participants; however, significant heterogeneity existed across the included studies. First, differences in study design: the included studies consisted of four RCTs, one quasi-RCT, and one single-arm study. The randomization bias inherent in the quasi-RCT and the uncontrolled design of the single-arm study precluded direct comparison of their results with those of the RCTs. Second, notable differences in population characteristics: the single-arm study enrolled younger women with a mean age of 33.7 years and a BMI of 36.4, whereas the other studies involved middle-aged populations with a mean age of 46 years and a BMI of 39.7. Younger individuals generally have higher metabolic rates and may experience less weight regain, while middle-aged individuals with obesity tend to exhibit more pronounced rebound, contributing to discrepancies in the results. Furthermore, the magnitude of weight regain after discontinuation of the same drug class varied across studies. For semaglutide, weight regain ranged from 6.9% to 11.6% across different studies, with significantly lower rebound observed in women with Polycystic Ovary Syndrome (PCOS) who received combination therapy with metformin. For tirzepatide, the SURMOUNT-4 study reported a 14% weight regain at 52 weeks after discontinuation, whereas real-world studies showed no difference in rebound in some patients, highlighting population specificity. Differences in interventions also led to bias in outcome measurements. The dosage of semaglutide was inconsistent: the study by Jensterle used 1.0 mg, while other studies used 2.4 mg. Similarly, the doses of tirzepatide (10 mg and 15 mg) were not uniform across studies. In addition, the follow-up duration ranged from 48 weeks to 2 years, representing a considerable span.

This study provides guidance for the clinical management of obesity and metabolic diseases. The differential weight regain following discontinuation of GLP-1RAs and GIP/GLP-1 dual agonists underscores the need for long-term maintenance therapy and individualized treatment strategies. Rosen & Ingelfinger (2026) explicitly noted in their review that although GLP-1RAs are highly effective for weight loss, weight regain after discontinuation remains a major challenge, requiring some form of maintenance therapy to prevent weight recovery; however, the specific type and duration of such therapy remain unclear. The meta-analysis by Berg et al. (2025) further quantified this issue, demonstrating that the extent of weight regain after discontinuation is proportional to the magnitude of initial weight loss, with a mean regain of 2.20 kg in the liraglutide group and 9.69 kg in the semaglutide/tirzepatide group. From a physiological perspective, the mathematical modeling analysis by Hubert et al. (2026) showed that weight loss with GLP-1 receptor agonist therapy reaches a peak of approximately 24% after about 96 weeks, followed by a plateau phase of approximately 78 weeks, during which the gap between energy intake and energy expenditure progressively narrows. This suggests that lower doses than those used during the initial treatment phase may be sufficient to maintain energy balance during the maintenance phase, providing a theoretical basis for low-dose maintenance strategies. The real-world study by Thomsen et al. (2025) further indicated that long-term weight maintenance strategies represent an important direction for future research, and that in current clinical practice, patients often use doses far lower than those evaluated in clinical trials. However, published clinical data specifically investigating low-dose GLP-1RAs for weight loss maintenance are still lacking.

Limitations of the existing studies also include short follow-up durations, with a lack of data beyond 5 years after drug discontinuation; the use of abrupt cessation protocols only, without exploration of gradual dose tapering; the low level of evidence in some real-world studies, with potential selection bias; and low GRADE scores for the primary outcomes, indicating that the current evidence is insufficient to mandate any specific discontinuation regimen. In addition, although the present study compared body weight at the time of drug discontinuation with that after discontinuation (see Supplementary Materials), direct comparison between post-discontinuation weight and pre-enrollment baseline weight is lacking; the latter would reflect the net benefit after completing treatment and subsequent discontinuation, which is of significant clinical relevance. Future studies should adopt high-quality RCT designs, avoiding quasi-RCTs and single-arm studies, to ensure comparability of results; establish clear inclusion criteria regarding age, BMI, sex, and the presence or absence of diabetes to avoid heterogeneity-induced variability; standardize GLP-1RA dosages to ensure consistency of interventions; ensure a follow-up duration of at least 1 year to guarantee comparability of outcomes; and conduct multicenter RCTs with larger sample sizes to enhance the reliability of the findings. Furthermore, clinical trials specifically designed to evaluate low-dose GLP-1RAs for weight loss maintenance should be conducted to fill the current evidence gap and provide clinically actionable maintenance dosing regimens.

## Conclusion

This meta-analysis, synthesising data from six studies involving 8,993 patients, confirms significant weight regain following discontinuation of GLP-1RAs and GIP/GLP-1 dual receptor agonists (discontinuation group *vs.* continued treatment group: MD = 17.90%, 95% CI [14.11–21.69]; post-discontinuation *vs.* pre-discontinuation: MD = 9.11%, 95% CI [7.91–10.30]). Subgroup analysis revealed a significantly greater rebound following discontinuation of tirzepatide compared with semaglutide (*P* = 0.0082), potentially attributable to compensatory metabolic disruption upon withdrawal of the dual receptor agonist mechanism and its shorter half-life. Overall and gastrointestinal adverse events decreased post-discontinuation, with no significant change in cardiovascular events. Current evidence is constrained by study heterogeneity and low GRADE quality ratings. Long-term follow-up multicenter studies are urgently required to optimise individualised maintenance therapy strategies and dose-reduction discontinuation protocols.

##  Supplemental Information

10.7717/peerj.21713/supp-1 Supplemental Information 1 PRISMA checklist

10.7717/peerj.21713/supp-2 Supplemental Information 2 basisc data

10.7717/peerj.21713/supp-3 Supplemental Information 3 Weight regain

10.7717/peerj.21713/supp-4 Supplemental Information 4 Any events

10.7717/peerj.21713/supp-5 Supplemental Information 5 Gastrointestinal disorders

10.7717/peerj.21713/supp-6 Supplemental Information 6 Weight Rebound Following Discontinuation Compared to Pre-Discontinuation

10.7717/peerj.21713/supp-7 Supplemental Information 7 Comparison of of body weight after discontinuation of medication versus prior to discontinuation

10.7717/peerj.21713/supp-8 Supplemental Information 8 Cardiovascular disorders

10.7717/peerj.21713/supp-9 Supplemental Information 9 ROB 2.0

10.7717/peerj.21713/supp-10 Supplemental Information 10 Weight regain before and after

## Additional Information and Declarations

### Data Availability

The following information was supplied regarding data availability: This is a Systematic review/Meta-analysis.
