# Supplementary material for: Weight regain following discontinuation of glucagon-like peptide-1 receptor agonists in adults who are overweight or obese: a systematic review and meta-analysis
Source: PeerJ. 2026 Sep 8;14:e21713. doi: 10.7717/peerj.21713 (PMC13564392; doi:10.7717/peerj.21713)
Supplement: Supplementary file 2 [file peerj-14-21713-s006.docx]

3.4.3 Weight Rebound Following Discontinuation Compared to Pre-Discontinuation

To assess the absolute extent of weight regain following discontinuation of treatment, we compared body weight at the end of the follow-up period with body weight at the time of discontinuation. Two studies [18, 22] reported these data. Calculations using a random-effects model showed that, compared with body weight at the time of discontinuation, body weight increased by an average of 9.11% (MD = 9.11%, 95% CI: 7.91–10.30, P < 0.0001); that is, patients’ body weight rebounded by 9.11% during the post-discontinuation follow-up period compared with immediately after discontinuation, as shown in Figure 5.
